# Recent History of *Aedes aegypti*: Vector Genomics and Epidemiology Records

**DOI:** 10.1093/biosci/biy119

**Published:** 2018-10-31

**Authors:** Jeffrey R Powell, Andrea Gloria-Soria, Panayiota Kotsakiozi

**Affiliations:** Yale University, in New Haven, Connecticut

**Keywords:** Aedes aegypti, history, yellow fever, dengue, slave trade

## Abstract

Aedes aegypti bears the common name “the yellow fever mosquito,” although, today, it is of more concern as the major vector of dengue, chikungunya, and, most recently, Zika viruses. In the present article, we review recent work on the population genetics of this mosquito in efforts to reconstruct its recent (approximately 600 years) history and relate these findings to epidemiological records of occurrences of diseases transmitted by this species. The two sources of information are remarkably congruent. Ae. aegypti was introduced to the New World 400–550 years ago from its ancestral home in West Africa via European slave trade. Ships from the New World returning to their European ports of origin introduced the species to the Mediterranean region around 1800, where it became established until about 1950. The Suez Canal opened in 1869 and Ae. aegypti was introduced into Asia by the 1870s, then on to Australia (1887) and the South Pacific (1904).

Regardless of whether *Aedes aegypti* is “the most dangerous animal in the world” (Powell 2016), there is little doubt that this mosquito has caused immense human suffering over centuries. Early in the twentieth century it was identified as the carrier of the yellow fever virus. Yellow fever ravaged the New World and doubtlessly affected major historical events that define the Americas today (McNeill 2010). More recently it has been the major vector of pandemics of three other viral diseases: dengue, chikungunya, and Zika fevers. It has been hypothesized that, of the approximately 3500 named mosquitoes, *Ae. aegypti* has been the single most important vector of these viruses because of a common evolutionary history in Africa along with the native African vertebrate host, humans (Powell 2018).

Given its importance to public health and human history, unraveling *Ae. aegypti*'s recent past (last 600 years) is important in order to understand how and when it came to occupy its present distribution, a distribution that determines human populations at risk for diseases it transmits. Not only will this help to understand historical disease patterns, it sheds light on threats this dynamic mosquito may pose in the future.

An obvious approach to this problem would be to simply consult the entomological literature for early reports of *Ae. aegypti* in different localities. Unfortunately, this is not possible as the taxonomy of *Ae. aegypti* (and many other mosquitoes) was rather muddled and unreliable until quite recently. For example, the medical team in Cuba working to identify the carrier of yellow fever called the mosquito they were using *Culex fasciata*, whereas there is little doubt it was *Ae. aegypti.* In fact, the original name, *Aedes aegypti,* was first used by Linnaeus in 1762 for a mosquito that was very likely not what is recognized today as *Ae. aegypti* (Mattingly 1957). Christophers (1960) summed up the problem: “The trouble was that it (*Ae. aegypti*) had so many aliases, almost one for every country and systematist.” The lack of consistency in use of species names, coupled with the absence of mosquito surveillance during the crucial period, 1500–1900, make such records unreliable, except in cases in which collections were deposited in museums and reexamined when nomenclature was clarified. Before approximately 1900 when it became known that mosquitoes transmit diseases, there was little reason for entomologists or public health workers to pay special attention to this little fly, except as a nuisance.

Two other, more reliable, sources of historical information are available. One is analysis of *Ae. aegypti* genomes in populations from different regions to trace back their history of connectedness, their phylogeography. Advances in population genomics have provided methods to estimate the number of generations since populations split and, by assuming generation times, this can provide estimates of dates of splits. A second source is to examine historical records of when and where diseases transmitted by *Ae. aegypti* are reported. This is possible for diseases that have distinctive symptoms recognizable before modern medical advances. For example malaria is clearly described in ancient Greece and Rome (Cunha and Cunha 2008). Yellow fever is similar: high fever, joint pain, and jaundice (yellowing of skin and whites of the eye) followed in later stages by black vomit resembling coffee grounds.

The purpose of this review is to illustrate how these two independent sources of information, genome analyses of mosquitoes and epidemiological history, mutually support a reasonably clear picture of the past 600 year history of *Ae. aegypti*.

## Origin of the domestic form

Multiple lines of evidence indicate that Africa is the ancestral home of *Ae. aegypti.* Populations still exist in forests of sub-Saharan Africa with tree holes and other natural pockets of water serving as larval breeding sites (Lounibos 1981). These populations prefer nonhuman mammals as a blood source (Gouck 1972; Peterson 1977; McBride et al. 2014). A major step in its history was when this “wild” species of mosquito became “domesticated,” i.e., began to use human-generated water containers for larvae and humans as a blood source. It is this domesticated form of *Ae. aegypti,* closely associated with and dependent on human habitats, that spread around the tropical and subtropical world and has been the source of worldwide epidemics of diseases it transmits (Powell and Tabachnick 2013). These two “forms” of *Ae. aegypti* have been given subspecies name, *Ae. aegypti formosus* for the ancestral African type and *Ae. aegypti aegypti* for the domestic type outside Africa. For convenience, we will use *Aaf* and *Aaa* to refer to these two forms.

When and how this wild species became domesticated has been the source of much speculation. One popular idea was that domestication was a consequence of the expansion of the Sahara Desert (Peterson 1977; Tabachnick 1991). Favorable tropical forest habitat for *Aaf* in Africa once extended as far north as the Mediterranean (Kropelin et al. 2008). It is hypothesized that, as the Sahara expanded 4000–6000 years ago, populations of *Ae. aegypti* isolated north of the nascent desert were forced to start breeding in the only reliable source of water remaining, that stored by humans in their towns and villages. This implies that *Ae. aegypti* was resident around the Mediterranean, at least on its southern shores, thousands of years ago. The Mediterranean could therefore be the source of the New World introductions (European trade after the “discovery” of the Americas) as well as Asian populations via frequent Mediterranean European–Asia trade over the centuries.

There are two problems with this scenario. First, had a domesticated form of *Ae. aegypti* existed in northern Africa thousands of years ago as a result of the expanding Sahara, it would almost certainly have spread around the Mediterranean as it did after 1800 (Schaffner and Mathis 2014, discussed later), clear evidence that the climate is favorable for establishment of this species in much of the Mediterranean Basin. Phoenician Carthage (in what is now Tunisia) carried out extensive trade around the entire Mediterranean for hundreds of years before being conquered by Rome in 146 BCE. However, unlike historical reports of malaria, there are no reports of yellow fever anywhere in the Mediterranean Basin before sporadic cases in and near seaports in the 1700s and self-sustaining epidemics in the 1800s (see later).

**Figure 1.**
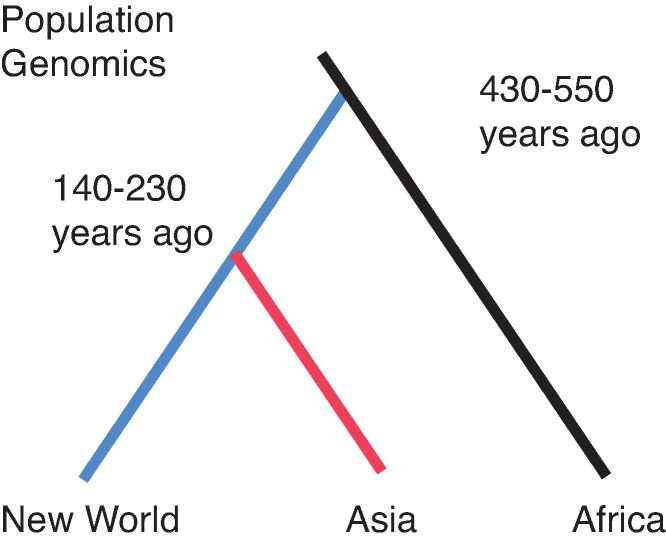
Graphical representation of the Aedes aegypti split of lineages and the times of events estimated from genetic data. The blue line represents the introduction into the New World with Asia (red line) being derived from the New World. There are three types of data: microsatellites (Gloria-Soria et al. 2016, Kotsakiozi et al. 2018) and exome sequences (Crawford et al. 2017). Two types of analyses to estimate times were used: approximate Bayesian computation analysis, as implemented in DIYABC (Cornuet et al. 2014), and allele array frequency analyses (Pickrell and Prichard 2012).

The second problem comes from genetic analyses of the mosquito. Genetic data strongly indicate that all domestic populations of *Aaa* outside Africa converged back to a single lineage, i.e., is monophyletic (Brown et al. 2014; Gloria-Soria et al. 2016; Kotsakiozi et al. 2018). Microsatellite data and DNA sequencing can now be used to estimate times when lineages split. Both data sets (Gloria-Soria et al. 2016; Crawford et al. 2017; Kotsakiozi et al. 2018) lead to estimates of 4000 to 5500 generations for this split. Assuming about ten generations per year for this mosquito in the tropics, this places the origin of domestic *Aaa* at 400–550 years ago (figure 1), much more recent than the expansion of the Sahara 4000–6000 years ago (Kropelin et al. 2008).

A more likely scenario is that *Ae. aegypti* became domesticated *in situ* in West Africa when human settlements began to form adjacent to forests. Much of West Africa experiences prolonged annual droughts when natural sites for *Ae. aegypti* larvae dry out. For example, Luanda in coastal Angola experiences a 6-month dry period (May–October) with an average of 5 millimeters of rain per month and less than 1mm for four of those months (June–September; Deutscher Wetterdienst 2016). Obviously, any human-occupied village would be storing water during the dry season. Although the eggs of *Ae. aegypti* remain viable when dried for a few months, by 4 months, hatching drops to about 1% with no hatching at 5 months (Christophers 1960). A female mosquito searching for a place to oviposit at the beginning of the dry season would find the stored water in villages very attractive. Females eclosing from such containers in the dry season would have no choice but to oviposit back into the human-generated water containers. They likely also evolve a taste for a new blood source: humans.

**Figure 2.**
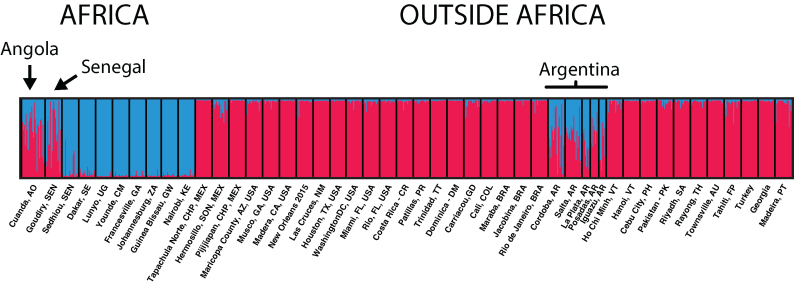
STRUCTURE plot of selected Ae. aegypti populations based on microsatellites (as implemented in Pritchard et al. 2000). Two clusters are defined (in red and blue), and the probability of assignment of individuals to the clusters is given by the colors of each line. All data are in Gloria-Soria and colleagues (2016), except for Turkey, Georgia, and Angola (which are in Kotsakiozi et al. 2018).

*Ae. aegypti* populations with mixed genetic signature typical of both the forest-breeding *Aaf* and domestic *Aaa* have been found in both Senegal and Angola (figure 2). These could represent the origins of the domestic lineage, what might be described as “proto-*Aaa*” populations (see supplement 1).

Although the two subspecies recognized today evolved morphological and behavioral differences, there is no indication of reproductive isolation between them (but see Dickson et al. 2016 for a possible exception). Historically, forests and villages were in sufficient proximity that the two forms continued to interbreed frequently enough to prevent evolution of reproductive isolation. It was not until the two types were entirely isolated from one another by the Atlantic Ocean that they evolved independently and “proto-*Aaa*” became fully *Aaa* we recognize today. The dating of the split of *Aaa* from *Aaf* using genetic data assumes complete isolation (no gene flow), so the 400–550 year estimate (figure 1) is when the two types stopped exchanging genes, after the ecological/behavioral divergence began within Africa (see supplement 2).

## Spread to the Americas

Populations of *Aaa* in the New World (North and South America) were the first to branch off from ancestral *Aaf* (figure 1). The time of this split (400–550 years ago) coincides with the rise of transatlantic shipping by Europeans. Beginning in the sixteenth century, ships originating in Europe would stop in West Africa to pick up native Africans for the slave trade before embarking on the crossing (Eltis and Richardson 2010). Doubtless they would also resupply with ample fresh water from coastal West African villages to last the 2–4 months needed to cross the Atlantic at that time. It is likely that eggs and larvae of *Ae. aegypti* would be included. Having already adapted to breeding in human-generated water storage containers preadapted these “proto-*Aaa*” mosquitoes to survive the long voyage.

The first reliable report of yellow fever in the New World was in 1648 in Havana and the Yucatan (McNeill 1976). Like the mosquito, the yellow fever virus is also native to Africa (Bryant et al. 2007; Powell 2018). This implies two things. First, by the mid-1600s, *Aaa* populations were established in high enough density to sustain yellow fever epidemics over a fairly wide area, at least in the Caribbean and Mexico. Second, slaves, sailors, and/or mosquitoes carrying the yellow fever virus must have survived the trip. The yellow fever virus remains infective in human carriers for only 7–10 days, adult mosquitoes seldom live more than a month, and transovarial transmission (vertical transmission in egg cytoplasm) of yellow fever virus is very rare (Aitken et al. 1979). This implies that multiple mosquito generations and transmission cycles occurred during the 2–4 months of the trip.

**Figure 3.**
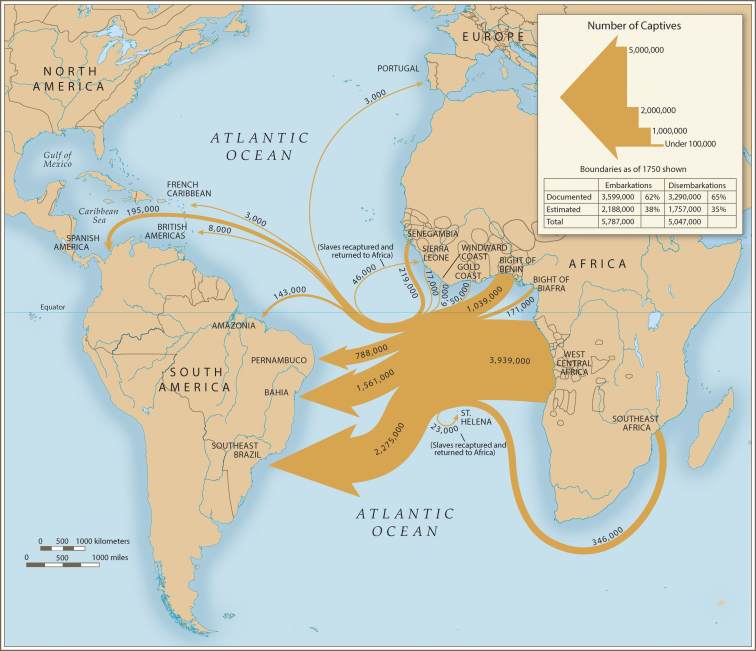
Quantitative depiction of Portuguese slave trade. The thickness of the arrows is proportional to the number of native Africans transported. From Eltis and Richardson (2010), used with permission.

Where in West Africa might the first introductions to the New World have originated? In the earliest days of European slave trade, 1500–1650, approximately 70% of the trade was carried out by Portugal (Eltis and Richardson 2010) and Portuguese ships almost exclusively used what is today Angola as their source of slaves (figure 3). An Angolan source is consistent with the genetic patterns although we cannot rule out other West African locales such as Senegal (figure 2).

Where was *Ae. aegypti* first introduced into the New World? Figure 3 suggests that Brazil or northern Argentina would be likely candidates. Because of a campaign to eliminate *Ae. aegypti* from the New World carried out between about 1950 and 1970, *Ae. aegypti* was eliminated from Brazil and subsequently recolonized from sources outside Brazil (Kotsakiozi et al. 2017). However, northern Argentina harbors populations very distinct from contemporary Brazilian populations that have genetic signatures of being closely related to *Aaf*, indistinguishable from the “proto-*Aaa*” populations in Angola and Senegal today (figure 2). No other populations outside Africa have such a genetic signature (Gloria-Soria et al. 2016; Kotsakiozi et al. 2018). These Argentine populations could represent relict populations that escaped the eradication program (see supplement 3). *Ae. aegypti* in Argentina have been reported to breed in tree holes (Mangudo et al. 2015), further evidence of retaining characteristics of their African ancestors.

## Back to the old world

Asia and the Pacific region (Australia and South Pacific Islands including Hawaii) have *Aaa* populations clearly derived from the New World (figure 1). How and when did *Aaa* get from the New World to Asia? European shipping may again hold the answer. The early Atlantic trade was triangular: starting in European ports, stopping in West Africa, proceeding to the New World, and returning to Europe. Just as these mosquitoes stowed away on the journey from Africa to the New World, they could well have been stowaways on the return trip to Mediterranean ports such as Spain and Portugal.

**Figure 4.**
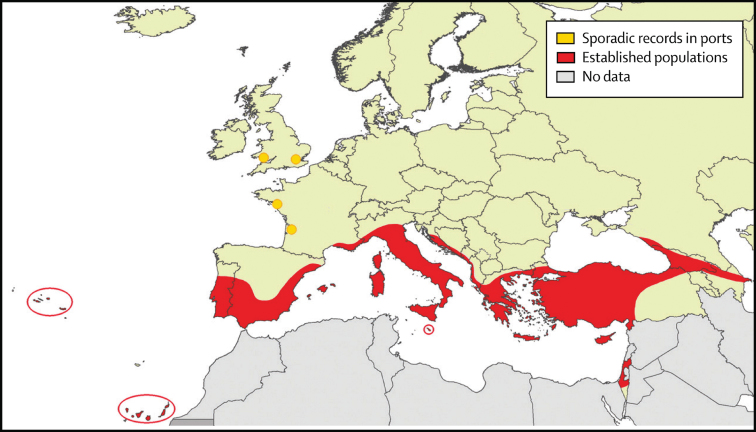
Historical distribution of Ae. aegypti in European countries before 1960. Northern Africa also had the species but is not depicted in this figure. Modified from Schaffner and Mathis (2014).

Although sporadic cases of yellow fever in Europe are reported in ports in which ships returned from the New World in the 1700s (Eager 1902), clear evidence of yellow fever transmission in Europe (autocthonous cases repeating annually) does not appear until 1800–1804, with severe outbreaks in several Spanish cities and Gibraltar resulting in more than 60,000 deaths (Augustin 1909; Sawchuk and Burke 1998). In letters written in 1801, the Queen of Spain describes suffering from a disease with symptoms typical of dengue (using the Spanish term *quebranta huesos,* break bone), despite never having been to the New World or Africa (Regau-Perez 1998). *Aaa* established itself around the Mediterranean (figure 4) and caused outbreaks of yellow fever and especially dengue in much of the Mediterranean throughout the nineteenth century and first half of the twentieth century. Greece was particularly hard hit in 1927–1928, with an estimated million cases of dengue and more than a thousand deaths (Rosen 1986). The time of establishment of Mediterranean populations around 1800 based on epidemiological data fits very well the population genomics data on the mosquito, assuming the now extinct Mediterranean populations were an intermediate between the New World and Asia, a “ghost taxon” in the tree in 1.

*Aaa* virtually disappeared from the Mediterranean around 1950 because of a combination of DDT application to control malaria-carrying Anopheles and, probably more importantly, improved sanitation and plumbing that eliminated the need to store water in houses, thus destroying this larval niche (Curtin 1967; Holstein 1967). Contemporary populations of *Aaa* around the Black Sea (Schaffner and Mathis 2014) could represent remnants of what once inhabited the Mediterranean. Although our recent genetic study of Black Sea *Aaa* indicated that these populations were ancestral to Asia, we could not unambiguously determine whether the Black Sea populations directly descended from the New World (Kotsakiozi et al. 2018).

## Asia/Australia/Pacific

The Suez Canal opened in 1869 and the first definitive cases of chikungunya in Asia appear in the 1870s (Carey 1971) and urban dengue in the 1890s (Smith 1956). Enigmatically, Asia has never experienced yellow fever, so presence of this distinctive disease cannot be used to time the introduction of *Aaa*. *Aaa*'s spread through Asia to the Pacific was rapid, probably because of the well-established trade routes in this part of the world by the late 1800s. The first reliable report of *Aaa* in Australia is in 1887, based on a museum specimen confirmed to be *Ae. aegypti* by modern workers (Lee et al. 1987). The first report of dengue in Australia is in 1897 (Hare 1898). [Interestingly, the *Ae. aegypti* specimen collected by Skuse in Brisbane in 1887 was called by him *Culex Bancroftii,* later changed to *Stegomyia fasciata* by Theobald in 1901 (Lee et al. 1987). This further highlights the ambiguity of nomenclature at that time.]

Australian *Aaa* today are genetically very close to S. Pacific populations that include the Philippines, Tahiti, and Hawaii (Gloria-Soria et al. 2016). The first reliable reports of *Ae. aegypti* on South Pacific Islands are from collections made in 1904 and called *Aedes argenteus* by Buxton and colleagues (Buxton 1927; Buxton and Hopkins 1927), later confirmed to be *Ae. aegypti*. Dengue did not arrive on the islands until the 1940s, speculated to have been introduced by troop movements during World War II (Monath 1994).

Formally, we cannot rule out the possibility that Asia/Australia/Pacific was colonized directly from the New World possibly from ships from the west coast of South America. Or there is what has been called the “First Fleet,” a group of 11 ships that left England in 1787 with 1100 convicts that stopped in Rio de Janeiro before traveling around the Cape of Good Hope to found penal colonies in Australia (Frost 2012).

However, the founding of Asia/Australia/Pacific directly from the New World without a Mediterranean intermediate is hard to reconcile with our observation that Black Sea populations are as old as, likely even older than, all contemporary Asia/Australia/Pacific populations (Kotsakiozi et al. 2018). This, coupled with the epidemiological data indicating the presence of *Aaa* in the Mediterranean before the opening of the Suez Canal and the appearance of *aegypti-*borne diseases in Asia very shortly after the opening, argues for the Mediterranean being an intermediate between the New World and Asia/Australia/Pacific.

## Conclusions

**Figure 5.**
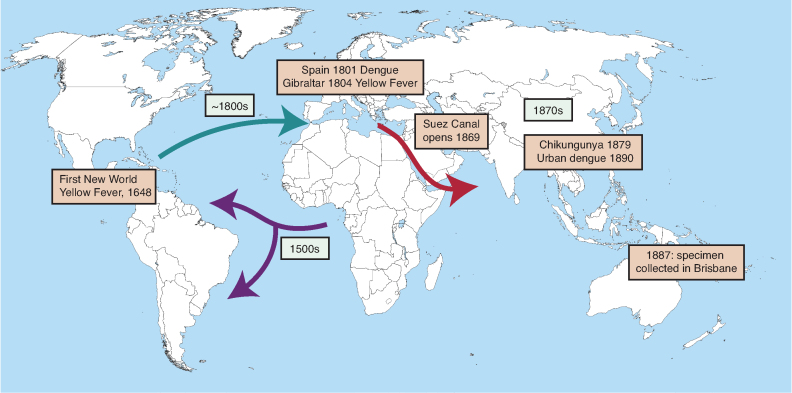
Summary of the history of Ae. aegypti over the last 600 years. The proposed routes of movement are shown by arrows with proposed dates. Major epidemiological events are noted in boxes. The times are consistent with dates of separation of mosquito populations estimated from genetic data (figure 1).

Figure 5 summarizes our present view of the last 600-year history of *Ae. aegypti.* Although speculation is involved in reaching our conclusions, we have presented a coherent hypothesis consistent with available data. The reinforcing nature of entirely independent types of data (mosquito genetics and epidemiologic history) lends credence to the hypothesis. However, this is a scientific hypothesis subject to falsification as further data arises.

## Supplementary Material

Supplemental data
