# Supplementary material for: Recent History of Aedes aegypti: Vector Genomics and Epidemiology Records
Source: Bioscience. 2018 Oct 31;68(11):854–60. doi: 10.1093/biosci/biy119 (PMC6238964; doi:10.1093/biosci/biy119)
Supplement: Supplemental data [file biy119_supplement_files.docx]

**SUPPLEMENTARY MATERIAL**

*BioScience*, August 2018

Recent History of *Aedes aegypti*: Vector genomics and epidemiology records.

Jeffrey R. Powell, Andrea Gloria-Soria, and Panayiota Kotsakiozi

**Supplement 1:** Are the mixed populations in some West African localities like Senegal and Angola (Figure 2) due to back migration of fully differentiated Aaa outside Africa or represent an intermediate evolutionary step prior to the founding of populations outside Africa? Our data could not unambiguously distinguish between these possibilities and we had initially interpreted the patterns observed as due to back migration (Brown et a. 2011; 2014; Gloria-Soria et a. 2016). The exome sequence data collected by Crawford et al. (2017) is more suited to address the *direction* of events leading to different genetic structures and their analysis favored gene flow from West Africa (Senegal) to the New World (Mexico) rather than the reverse. Because this is the only data-based test of this issue to date, we tentatively accept this scenario as the best interpretation given present knowledge. This implies that the mixed West African populations are an intermediate between full Aaa and Aaf, what we call “proto Aaa”.

**Supplement 2:** Crawford et al. (2017) estimate that the domestication within Africa may have started as far back as 16,000 years, although our data would suggest a much shorter time. Climatic data analysis (Wanner et al. 2008) provide evidence for “a pronounced weakening of the monsoon systems in Africa and Asia and increasing dryness and desertification on both continents” about 2-4,000 years ago. Ice core analyses indicate the “greatest historically recorded drought in tropical Africa” occurred about 4,000 years ago. Just when this domestication started must remain an open question, but it certainly pre-dated the ~500 year old origin of New World populations that led to the fully differentiated Aaa we find today.

**Supplement 3:** In addition to our data, Paupy et al. (2012) and Llinas et al. (2012) interpreted results from analysis of variation in mtDNA in *Ae. aegypti* from this region of S. America (northeast Argentina/southern Bolivia) as indicative of relict populations that escaped the eradication program of 1950-70. Roberts (2016) presents a map indicating *Ae. aegypti* in this region after it had been eradicated from most of the continent including Brazil. Alternatively, these mixed Argentina populations could represent a recent introduction of Aaf (as we initially thought, Gloria-Soria et al. 2016). However, the arguments in Supplement 1 indicating that genetically similar populations (Figure 2) were the founders of the New World, makes the interpretation of Argentina harboring relict populations stronger. While *Ae. aegypti* outside Africa is known to oviposit in natural pools of water (e.g., Chadee et al. 1998), this most often occurs on islands with a limited mosquito fauna so there are no native competitors in the natural sites. In tropical mainland South America there are many native tree hole species that make this site difficult for fully domesticated *Ae. aegypti* to exploit.

**Reference for supplements:**

Chadee DD, Ward RA, Novak RJ 1998. Natural habitats of *Aedes aegypti* in the Caribbean – a review. Journal of the American Mosquito Control Association 14: 5-11.

Llinas GA, Gardenal CM. 2012. Phylogography of *Aedes aegypti* in Argentina: long-distance colonization and rapid restoration of fragmented relicts after a continental control campaign. Vector-borne and Zoonotic Dis. 12:254-261.

Paupy C, Le Goff G, Brengues C, Guerra M, Revollo J, Simon ZB, Herve J-P, Fotenille D. 2012. Genetic structure and phylogeography of *Aedes aegypti,* the dengue and yellow-fever mosquito vector in Bolivia*.* Infections, Genetics and Evolution 12:1260-1269.

Roberts, D. 2016. Virus infections in the Americas and the DDT question. American Council Science and Health  https://www.acsh.org/news/2016/02/23/zika-virus-infections-in-the-americas-and-the-ddt-question

Wanner, H., J. Beer, J. Butikofer, et al. 2008. Mid- to late Holocene climate change: and overview. Quarternary Science Reviews 27:1791-1828.

Thompson, L. G., E. Mosley-Thompson, M. E. Davis, K. A. Henderson, H. H. Brecher, V. S. Zagorodnov, T. A. Mashiotta, P.-N. Lin, V. N. Mikhalenko, D. R. Hardy, and J. Beer. 2002. Evidence of Holocene climate change in tropical Africa. Science 298: 589-593.
